# Biophysical Characterization and Membrane Interaction of the Two Fusion Loops of Glycoprotein B from Herpes Simplex Type I Virus

**DOI:** 10.1371/journal.pone.0032186

**Published:** 2012-02-23

**Authors:** Annarita Falanga, Rossella Tarallo, Giuseppe Vitiello, Mariateresa Vitiello, Emiliana Perillo, Marco Cantisani, Gerardino D'Errico, Massimiliano Galdiero, Stefania Galdiero

**Affiliations:** 1 Division of Biostructures, Department of Biological Sciences, University of Naples “Federico II”, Napoli, Italy; 2 Centro Interuniversitario di Ricerca sui Peptidi Bioattivi, University of Naples “Federico II”, Napoli, Italy; 3 Department of Chemistry, University of Naples “Federico II” and Consorzio per lo Studio dei Sistemi a Grande Interfase, CSGI, Monte Sant'Angelo, Napoli, Italy; 4 Department of Experimental Medicine, II University of Naples, Napoli, Italy; 5 Istituto di Biostrutture e Bioimmagini, CNR, Napoli, Italy; University of Minnesota, United States of America

## Abstract

The molecular mechanism of entry of herpesviruses requires a multicomponent fusion system. Cell invasion by Herpes simplex virus (HSV) requires four virally encoded glycoproteins: namely gD, gB and gH/gL. The role of gB has remained elusive until recently when the crystal structure of HSV-1 gB became available and the fusion potential of gB was clearly demonstrated. Although much information on gB structure/function relationship has been gathered in recent years, the elucidation of the nature of the fine interactions between gB fusion loops and the membrane bilayer may help to understand the precise molecular mechanism behind herpesvirus-host cell membrane fusion. Here, we report the first biophysical study on the two fusion peptides of gB, with a particular focus on the effects determined by both peptides on lipid bilayers of various compositions. The two fusion loops constitute a structural subdomain wherein key hydrophobic amino acids form a ridge that is supported on both sides by charged residues. When used together the two fusion loops have the ability to significantly destabilize the target membrane bilayer, notwithstanding their low bilayer penetration when used separately. These data support the model of gB fusion loops insertion into cholesterol enriched membranes.

## Introduction

Enveloped viruses infect host cells by fusion of viral and target membranes. The initial apposition step is followed by fusion of the outer leaflets of membranes (the hemifusion step), leading to the formation of a transient fusion intermediate, which evolves into the fusion of inner leaflets and the formation of a pore, with the mixing of the internal compartments of both fusion partners. Thus, the viral genome is transferred to the cytoplasm of the host cell and viral replication ensues [1]–[3]. This fusion event is triggered by specific glycoproteins in the viral envelope. Fusion glycoproteins belong to either class I, class II or the newly described class III, depending upon their arrangement at the surface of the virion, their structure and the location within the protein of a short stretch of hydrophobic amino acids called the fusion peptide, which is able to induce the initial lipid destabilization that ends in fusion [4].

Although, viral fusion proteins are divided in three classes and the cellular membrane undergoing fusion might vary (plasma membrane vs. endosomal membrane), these proteins have to act on lipid assemblies. Thus, lipids contribute to fusion through their physical, mechanical and/or chemical properties and play a role in determining the preferential partitioning of some amino acid sequences into membrane microdomains called “rafts”, and/or in modulating the curvature of the membranes involved in the fusion process [3].

Considering lipids and fusion proteins the necessary partners involved in the fusion process, it becomes clear that the overall gist of viral-induced membrane fusion is dictated by several features common to different families of viruses [1]. All fusion machineries need to interact with lipids, so they possess hydrophobic segments (fusion peptides) or are able after rearrangements to make hydrophobic interactions with membranes; moreover, need to adopt specific conformations related to the pre and post-fusion states, since fusion is limited in space and time. Therefore, different viruses infect their host cells by very similar mechanisms at the molecular level and fusion peptides represent a key element of the fusion machinery, being the trigger for controlled membrane destabilization.

Recently, the novel concept of “lipid packing sensor” emerged, indicating protein motifs or domains that recognize the curvature of lipid membranes. The most studied sensor is the amphipathic helical motif present both in cellular membrane proteins and in viral proteins [5], [6]; but also other structural motifs, such as loops or unfolded peptides, may be involved in the sensing of the membrane curvature and in the initial steps of membrane fusion. The discovery of sensor motifs opens new perspectives with respect to viral fusion proteins, which may contain such sensors; and may help our understanding of the subtle and complex interplay between protein-induced fusion and lipid modulation and may represent targets for future antiviral therapies.

Herpes simplex virus type 1 (HSV-1) is a member of the α-herpesvirus subfamily and enters cells through fusion of the viral envelope with a cellular membrane in a cascade of molecular interactions involving multiple viral glycoproteins and cellular receptors. The envelope glycoproteins gH/gL, gB and gD are all essential for the entry process [7] and expression of this quartet of glycoproteins induces the fusion of cellular membranes in the absence of virus infection [8]. gH/gL and gB, also play important roles in primary fusion events that occur during egress of the capsid from the nucleus of infected cells [9]. Recently, it was shown that gB and gH/gL interact with each other concomitantly with fusion and that this interaction is triggered by binding of gD to its cellular receptor [10], [11]. Thus, gB may function cooperatively with gH/gL, and each may have some fusogenic potential on its own. Although gB and gH/gL constitute the core fusion machinery of all members of the Herpesviridae, their mechanisms of action are still under investigation.

Peptides derived from the ectodomain of gH block virus entry [12], while others have the ability to bind and disrupt model membranes [13]–[18]. The recently solved crystal structure of the gH-gL complex [19] showed that gH has no structural homology with any known fusion protein supporting the hypothesis that gH may act as a regulator of fusion through interactions with gB, while the crystal structure of the gH-gL complex of EBV presents considerable differences in the structural arrangements of domains supporting the view that the complex can undergo dynamic rearrangements [7], [20].

gB is highly conserved within all the members of the Herpesviridae and is involved in virus attachment, penetration and cell-to-cell spread. gB may undergo large conformational changes to bring about fusion and even though evidence for its refolding has yet to be obtained a model for pre-fusion gB has been proposed and it seems that gB undergoes a refolding transition during fusion. The structures of gB from HSV-1 [21] and EBV [22] demonstrated that gB has structural homology with viral fusion proteins belonging to other virus families: the vesicular stomatitis virus (VSV) G protein [23] and the baculovirus protein gp64 [24] and is probably the key fusion protein; moreover, several synthetic gB peptides induced the fusion of large unilamellar vesicles and inhibited herpes virus infection [25], [26]. gB belongs to a newly formed group of fusion proteins, class III, which share similar individual domain structures and contain a central three-stranded coiled-coil reminiscent of the class I proteins. Whereas class I proteins have an N-terminal fusion peptide, class III proteins have internal bipartite fusion loops within domain I which are similar to the single fusion loop of class II fusion proteins. The class II fusion loop is composed entirely of hydrophobic amino acids whereas those of gB have both hydrophobic and charged residues [21]–[24]. In particular, the fusogenic loops correspond to the domains 173–179 and 258–265 of the protein gB.

Hydrophobic amino acids within the gB loops (W174, F175, G176, Y179, A261) are essential for gB function [27]. Similar studies of VSV G, gp64 and EBV gB support the notion that hydrophobic amino acids of both fusion loops are critical for fusion [28], [29] and together constitute a fusion domain. Moreover, also charged amino acids play a fundamental role, in fact, mutations of H263 or R264 also negatively affected gB function [27]. These data support the view that the two fusion loops constitute a subdomain where key hydrophobic amino acids form a ridge that is flanked on both sides by charged amino acids that enhance the ability to interact with target membranes, in fact, one of the two gB fusion loops (namely HB168–186) was identified by highly interfacial hydrophobicity analysis without the aid of structural data [25].

Although much information has been gathered in recent years, we do not yet know the precise molecular mechanism behind herpesvirus host cell membrane fusion; thus, elucidating the nature of the fine interactions between gB fusion loops and the membrane may help to districate this interesting matter. In the present study, we report the synthesis and characterization of membrane interactions of the two fusion peptides present in gB. The goals of the experiments reported here were to verify the fusion ability of the two peptides, to assess the dependence of the fusion activity on the composition of the membrane and in particular on the content of cholesterol and to verify the eventual presence of a synergic or cooperative effect when the two isolated peptides are used together.

As far as we know, this is the first biophysical study on the two putative fusion peptides of gB, with a particular focus on the effects determined by both peptides on lipid bilayers of various compositions.

## Results

### Design of peptides

**Figure 1:**
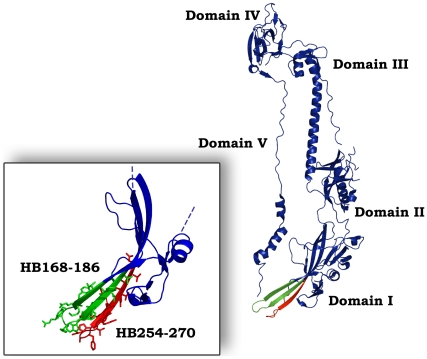
Three dimensional structure of a single gB protomer. The different domains are shown. In the insert are shown molecular details of the two fusion loops: HB168–186 (green) and HB254–270 (red).

The proposed post-fusion structure of gB ectodomain is comprised of three protomers, and each protomer coils around the others with a left-handed twist [21]. There are multiple contacts between protomers throughout the molecule contributing to trimer stability. Each protomer of gB can be divided into five distinct domains: I, base; II, middle, III, core; IV crown; and V, arm (Figure 1). Domain I (Ile154 to Val363) is a β sandwich composed of two nearly orthogonal β sheets of four and three strands, with a long loop and short helix covering one opening of the β sandwich. An insertion (Tyr165 to Ile272) between strands β4 and β11 creates a subdomain at the base of the trimer, consisting of a four-stranded β sheet (with three long and one short strand), the convex side of which is covered with an α helix, a β hairpin, and a short two-stranded β sheet; the four-stranded β-sheet presents hydrophobic tips that have been proposed to represent the fusion peptides of gB (Figure 1). Fusion peptides are generally considered as being domains with a high interfacial hydrophobicity calculated with the Wimley-White hydrophobicity scale which was systematically used for searching fusion peptides in other viral glycoproteins. HB168–186, comprising the precise fusion loop HB173–179 [21], is located in the insertion between strands β4 and β11 and we were able to identify this region on the basis of its hydrophobicity, and analyse its role in peptide/lipid fusion assays [25]. The second loop (HB258–265), identified on the basis of structural data [21] contributes to the formation of the bipartite fusion peptide, but does not correspond to a hydrophobicity peak calculated with the interfacial scale and thus was not considered in our previous study [25].

Both fusion loops have been characterized by point-mutations in the protein sequence and showed to be critical for gB function in cell fusion [30]. In the present study, we report on the structural characterization of the two fusion loops by using peptides corresponding to their sequences. We synthesised a short version, corresponding exactly to the sequences of the loops, and a longer version, embracing the flanking β-sheets, in order to understand the contributions of each region that constitutes the gB fusogenic domain (Table 1). Moreover, we tried to understand the contribution of lipids that take part to the interplay between the membrane and fusogenic peptides.

**Table 1:** Peptides.

	Sequence
**HB168–186**	NH_2_-VTVSQVWFGHRYSQFMGIF-COOH
**HB173–179**	NH_2_-VWFGHRY-COOH
**HB254–270**	NH_2_-YNPSRVEAFHRYGTTVN-COOH
**HB258–265**	NH_2_-RVEAFHRY-COOH
**scrambled**	NH_2_-MRWFSVVSYQVIGTQFGFH-COOH

### Ability of peptides to induce lipid mixing

To investigate the fusogenicity of gB peptides, NBD and Rho labelled PE were used as the donor and acceptor of fluorescence energy transfer. A population of LUVs labelled with both was mixed with a population of unlabelled LUVs and increasing amounts of peptides were added. Dilution of the fluorescent-labelled vesicles via membrane fusion induced by the peptide results in a reduction of the fluorescence energy transfer efficiency, hence dequenching of the donor fluorescence. The dependence of the extent of lipid mixing on the peptide to lipid molar ratio was analysed. The fusogenic capacity was monitored by measuring their ability to induce lipid mixing of model membranes composed of DOPG, DOPG/Chol (3/2), DOPC, DOPC/Chol (3/2), POPC/Chol (3/2), and POPC/Chol/SM at various ratios (4/1/1, 2/1/1, 1/1/1).

**Figure 2:**
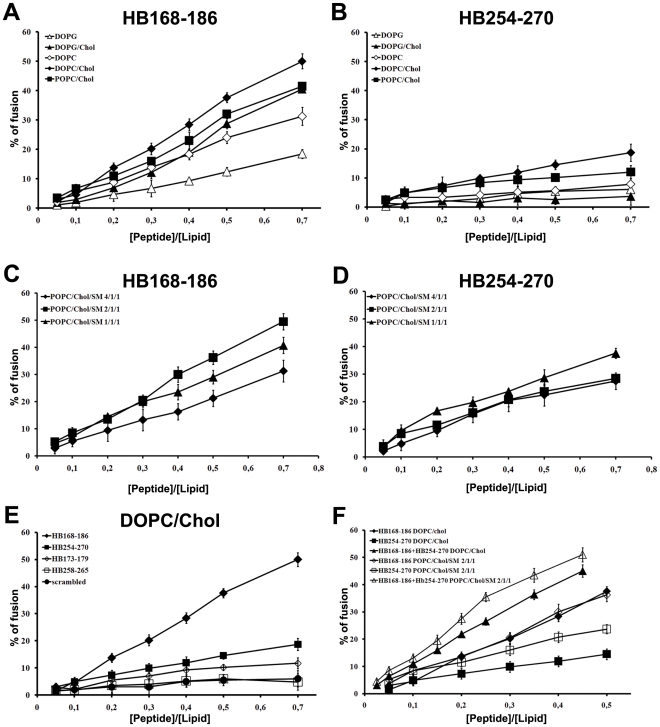
Ability of peptides to induce lipid mixing. Peptide-promoted membrane fusion as determined by lipid mixing; peptide aliquots were added to 0.1 mM LUVs, containing 0.6% NBD and 0.6% Rho. The increase in fluorescence was measured after the addition of peptide aliquots; reduced Triton-X-100 (0.05% v/v) was referred to as 100% of fusion. In figure is reported the dose dependence of lipid mixing at 37°C: panels A and B HB168–186 and HB254–270 in LUVs of different composition; panels C and D HB168–186 and HB254–270 in LUVs mimicking lipid rafts; panel E HB168–186, HB254–270 and shorter versions in DOPC/Chol LUVs; panel F equimolecular mixtures of peptides HB168–186 and HB254–270 in DOPC/Chol (3/2) and POPC/Chol/SM (2/1/1).

Interestingly, there was a different behaviour of the peptides depending on the lipid composition of LUVs. Figure 2A and B show the results of lipid mixing assays in LUVs of different compositions for peptides HB168–186 and HB254–270. The graphs show that in all tested conditions HB168–186 has higher fusogenic ability. DOPC/Chol, POPC/Chol and DOPG/Chol all proved to be good fusogenic conditions. Furthermore, it is evident from the results obtained that DOPG in presence or absence of cholesterol represents a less fusogenic condition, indicating that the negatively charged phospholipids are not preferred. This is a rather interesting result as both peptides contain arginine residues and thus we could expect a greater interaction with anionic lipids. Several viruses such as HIV, influenza virus and Semliki Forest virus have been shown to involve lipid microdomains, which are enriched in cholesterol during virus budding as well as virus entry. Also herpesvirus has been shown to require cholesterol [31], [32], and such a need for cholesterol has also been highlighted in our experiments using model membranes where a significant increase in fusion is always observed in presence of cholesterol. The lipids allowing the highest level of fusion are DOPC and POPC which are both zwitterionic but present a difference in their unsaturation, with POPC having only one unsaturated acyl chain and DOPC having two. DOPC results in a more flexible bilayer, which appears to be an important feature for the fusion activity of the analyzed peptides. We also tested the peptide fusion ability of liposomes containing sphingomyelin, a component commonly found in lipid rafts. The ternary lipid system POPC/Chol/SM is a good model for lipid rafts and from the ternary phase diagram [33] it is possible to determine the boundaries for lipid rafts which are strongly dependent on the percentage of each lipid in the system. We used three different conditions to probe the activity of our peptides in LUVs mimicking rafts: POPC/Chol/SM 4/1/1, 2/1/1 and 1/1/1, characterized by different percentages of the liquid ordered and liquid disordered phases. The results obtained (Figure 2 C–D) show that both peptides present a significant percentage of fusion

Figure 2E shows the results obtained in DOPC/Chol for the peptides HB168–186 and HB254–270 as well as their shorter versions corresponding exactly to the loop sequences derived from the crystallographic structure. The two shorter peptides (HB173–179 and HB258–265) induced lower levels of fusion compared to longer sequences, suggesting that shorter peptides were unable to cross or stably position inside the bilayer. This result further supports the view that fusion loops are structured by the overall organization of the fusion domain of the protein and longer peptides are, therefore, necessary to evidence an appreciable fusion activity.

In the case of the peptide equimolar mixtures (Figure 2F), we can observe that in DOPC/Chol as well as in POPC/Chol/SM (2/1/1) we have a significant fusion activity supporting a cooperative mechanism.

### Ability of peptides to induce lipid mixing of inner-monolayer

**Figure 3:**
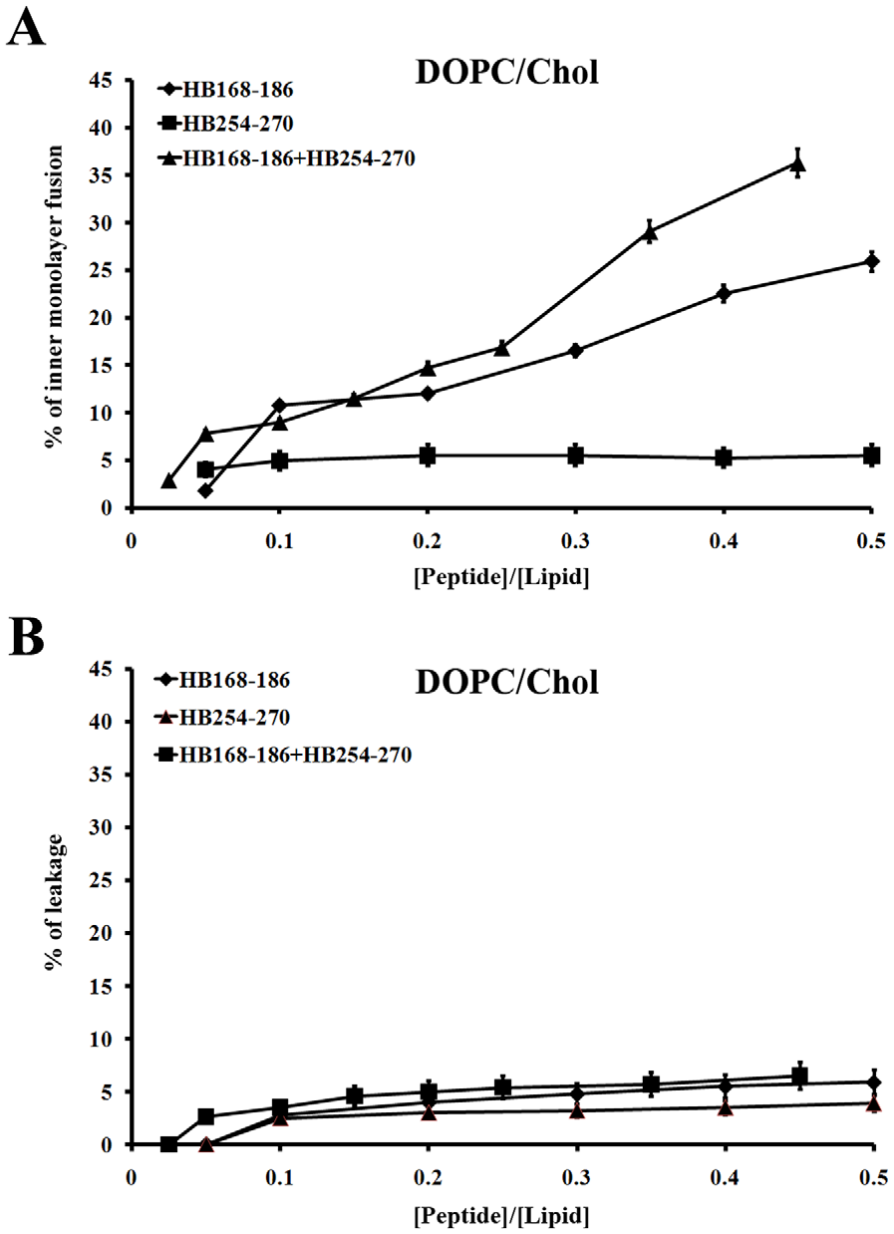
Ability of peptides to induce lipid mixing of inner-monolayer and membrane leakage. Peptide interactions with DOPC/Chol LUVs (A–B), the dose dependence is reported and each trace represents an average of three independent experiments. A) Inner monolayer assay. C)Leakage of ANTS/DPX.

In the inner monolayer assay, the fluorescence from the vesicle membranes' outer monolayer is eliminated by the addition of an aqueous reducing agent, and this experiment reveals the extent of lipid mixing between the inner monolayers of vesicles in solution. Figure 3A shows a significant fusion of the inner monolayer in DOPC/Chol. This is slightly lower than the fusion level obtained in the lipid mixing experiment, since the latter measures both hemi-fusion and complete fusion. Therefore, this assay clearly indicates that the two peptides are able to induce fusion of both the inner and the outer monolayers.

We also verified the ability of peptide equimolar mixtures to induce lipid mixing of the inner-monolayer. The results obtained indicate that the peptides cooperate in the fusion process.

### Ability of peptides to induce membrane leakage

In order to explore the effects of the peptides in the destabilization of membrane vesicles, we studied their effect on the release of encapsulated fluorophores in model membranes made of DOPC/Chol. A content-mixing assay (Figure 3B) was employed to monitor any mixing of internal vesicle components as a result of vesicle exposure to HB168–186 and HB254–270. Release of ANTS and DPX from vesicle is commonly used as a measure of bilayer perturbation and interpreted as “transient pore formation” [34], [35]. Content-mixing is manifested by a decrease in fluorescence intensity if vesicles encapsulating fluorescent cargo (e.g., ANTS) merge contents with those containing quenchers (e.g., DPX). The leakage experiment shows that the probe did not leak out significantly to the medium after the interaction with any of the peptides used in this study. Figure 3B shows that no content-mixing occurs over the same P/L range where substantial outer and inner monolayer lipid-mixing occurs, confirming that vesicle fusion may happen within our system without concomitant pore formation. The low leakage value observed for both peptides might be due to the fact that they are located at the membrane interface and do not completely traverse the bilayer. We also verified the eventual ability of equimolar mixtures of HB168–186 and HB254–270 to induce membrane leakage, but no significant difference from results obtained for individual peptides was detected.

### Peptide aggregation

**Figure 4:**
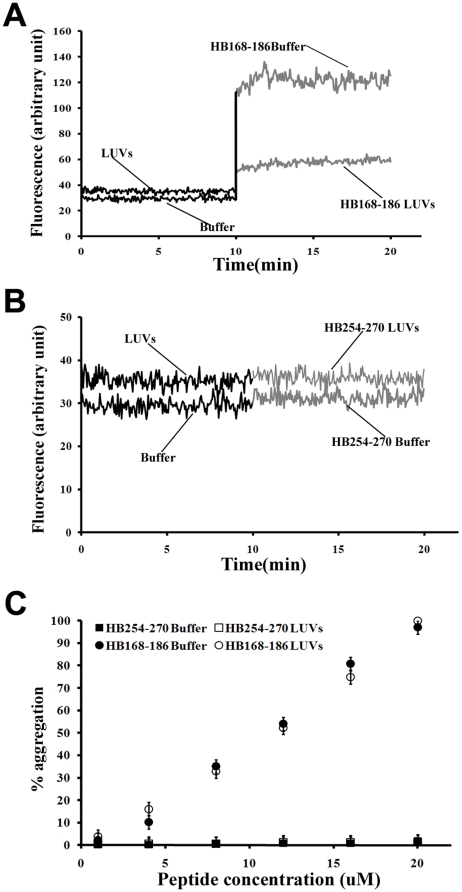
Peptide aggregation. Fluorescence variation of ThT after addition of HB168–186 (A) and HB254–270 (B) peptides to an aqueous solution and in the presence of LUVs of DOPC/Chol at a peptide concentration of 4 µM. (C) Percentage of aggregation as a function of peptide concentration for HB168–186 and HB254–270 in buffer (closed symbols) and in LUVs (open symbols).

The peptide's aggregation state in buffer and DOPC/Chol LUVs was assayed using ThT [36], in order to know the possible effects of the peptide aggregation on the membranes. As observed in Figure 4 A–B, the two peptides present a completely different behaviour. The peptide HB168–186 at a concentration of 4 µM (Figure 4A) is already aggregated in an aqueous medium, in fact, the fluorescence change increased dramatically after the addition of the peptide from a stock solution, while in the presence of DOPC/Chol a lower aggregation is reported. The peptide HB254–270 is not aggregated in the aqueous medium nor in the presence of DOPC/Chol at the same concentration. Furthermore, we studied the intrinsic relationship between the concentration of the peptides and the aggregation in buffer and in LUVs and we can interpret the data for HB168–186 reported in Figure 4C as aggregated both in the aqueous medium and in LUVs at high concentrations. Our results are consistent with two hypothesis; the first is an initial aggregation in the aqueous medium, followed by a disaggregation when initially interacting with the membrane and again an aggregation inside the membrane bilayer, indicating that the insertion of the peptide into the membrane could occur in a monomeric form and only afterwards the peptide aggregates again; while the second hypothesis is that the peptide can interact with the membranes already in an oligomerized state.

### Tryptophan/tyrosine fluorescence emission analysis

We measured the intrinsic fluorescence of HB168–186 and HB254–270 (due to the presence of a tryptophan residue in the sequence of HB168–186 and two tyrosines for HB254–270) to evaluate the degree of penetration of the peptides into the membrane bilayer. We compared the fluorescence emission spectra in DOPC/Chol vesicles with that in buffer (data not shown). The quantum yield of aromatic residues of a peptide or protein normally changes when the amino acid is located in a more hydrophobic environment such as a phospholipid membrane, normally increasing the intensity of the fluorescence emission and shifting the maximal spectral position toward shorter wavelengths (blue shift). Changes in the spectral properties were observed for both peptides, suggesting that the single tryptophan residue of HB168–186 and the two tyrosines of HB254–270 move to a less polar environment upon interaction with lipids. Emission intensity was enhanced and the maxima shifted to lower wavelength. (Figure S1) Blue shifts of this magnitude are generally observed when amphiphilic aromatic-containing peptides interact with phospholipid bilayers and are consistent with the aromatic moiety becoming partially immersed in the membrane, further suggesting that the analysed peptides are capable of penetrating a lipid bilayer [37].

**Figure 5:**
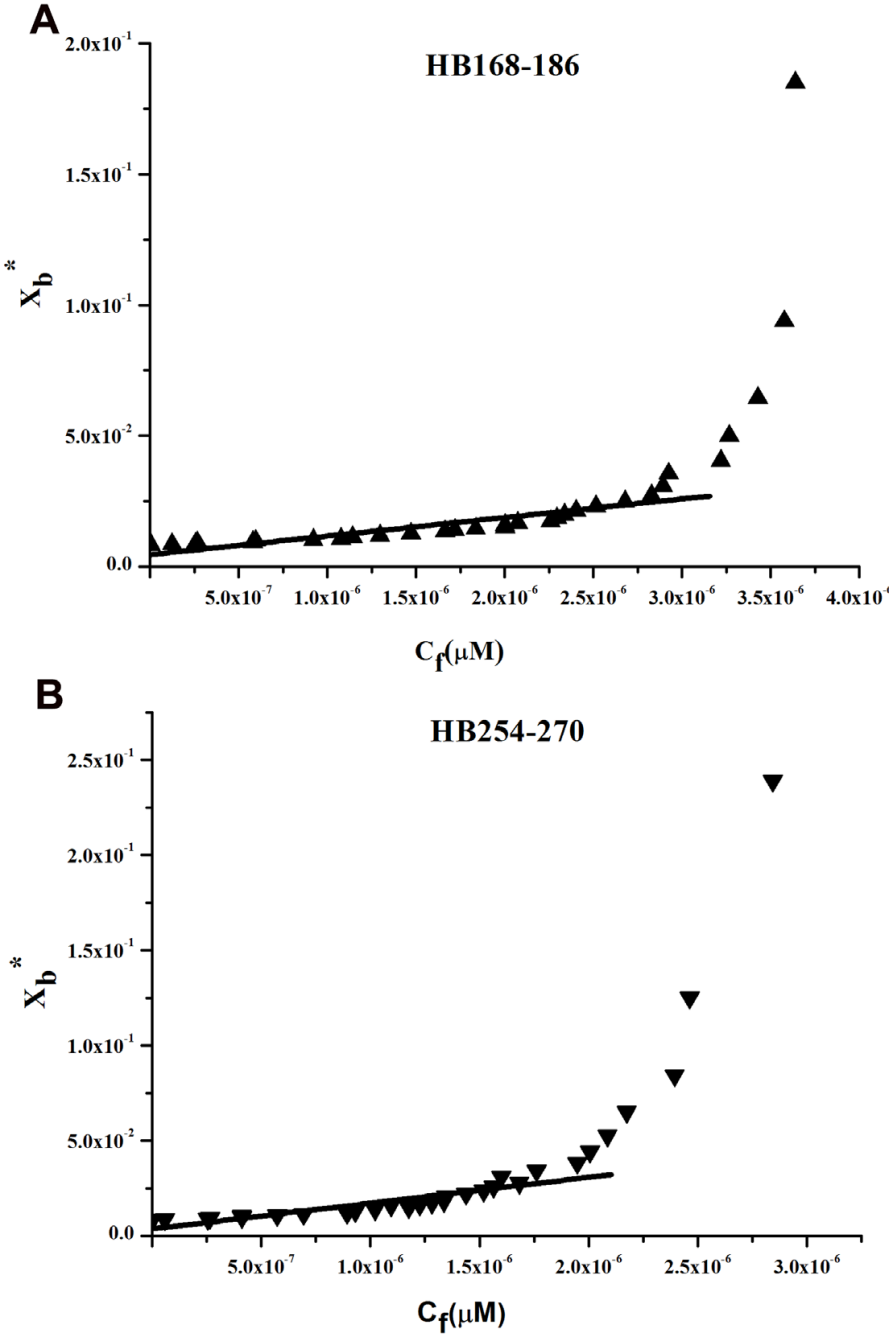
Tryptophan/tyrosine fluorescence emission analyses. Binding isotherms obtained plotting X_b_^*^ versus C_f_ for HB168–186 and HB254–270.

The increase in fluorescence for tryptophan or tyrosines binding to membrane phospholipids was used for the generation of binding isotherms for HB168–186 and HB254–270, therefore partition coefficients could be calculated. The concentrations of peptides used were low enough to cause minimal aggregation in the aqueous phase and were assumed not to disrupt the bilayer structure. To determine the surface partition coefficient, the fluorescence intensities were converted to moles of bound peptide per moles of lipid and plotted as a function of the free peptide concentration as described in material and methods (Figure 5). As partition coefficients depend on the concentration of lipid accessible to peptide, the curves obtained by plotting *X*_b_* (the molar ratio of bound peptide per 60% of the total lipid) vs *C*_f_ (the equilibrium concentration of free peptide in the solution) are referred to as the conventional binding isotherms. The shape of a binding isotherm of a peptide can provide information on the organization of the peptide within the membrane. A straight line indicates a simple adhesion process. The shape of the binding isotherm of all the peptides tested was not linear indicating that peptide accumulation at the surface is not a simple phenomenon without cooperative association. In particular, this behaviour is the hallmark for peptides that self-associate at membrane surfaces upon partitioning. If aggregation occurred only in the water but not in the bilayer phase, the opposite course of the isotherms should be expected: a steep rise at the origin, followed by pronounced flattening; thus, the shape of the isotherms obtained could be interpreted as reflecting a process whereby peptides first incorporate into the membrane and then aggregate there within. Moreover, there was no evidence of significant aggregation in water at the concentration used in this experiment (0.1 µM) for HB254–270, although we have an indication of aggregation in water for HB168–186 as shown in Figure 4C. In the isotherms obtained, the total extent of incorporation (X_b_^*^) slowly increases until a critical concentration is reached, where massive internal aggregation apparently starts to develop.

The surface partition coefficients K_p_ were estimated by extrapolating the initial slopes of the curves to *C*_f_ values of zero. Curves are shown in Figure 5.

The K_p_ values for the peptides HB168–186 and HB254–270 are shown in Table 2. The K_p_ value obtained for HB168–186 is 2.5 10^4^, indicating that the tryptophan in HB168–186 is able to interact significantly with the bilayer and that most of the peptide HB168–186 is located inside the liposomes. The K_p_ value for HB254–270 is 3.2 10^4^, indicating that the tyrosine residues present in this peptide are located inside the liposomes and are stably inserted.

**Table 2:** Partition coefficient for the binding of peptides with PC/Chol.

	HB168–186	HB254–270
**K_p_**	(2.5±0.3)10^4^	(3.2±0.2)10^4^

### Quenching of tryptophan/tyrosines by Acrylamide

**Figure 6:**
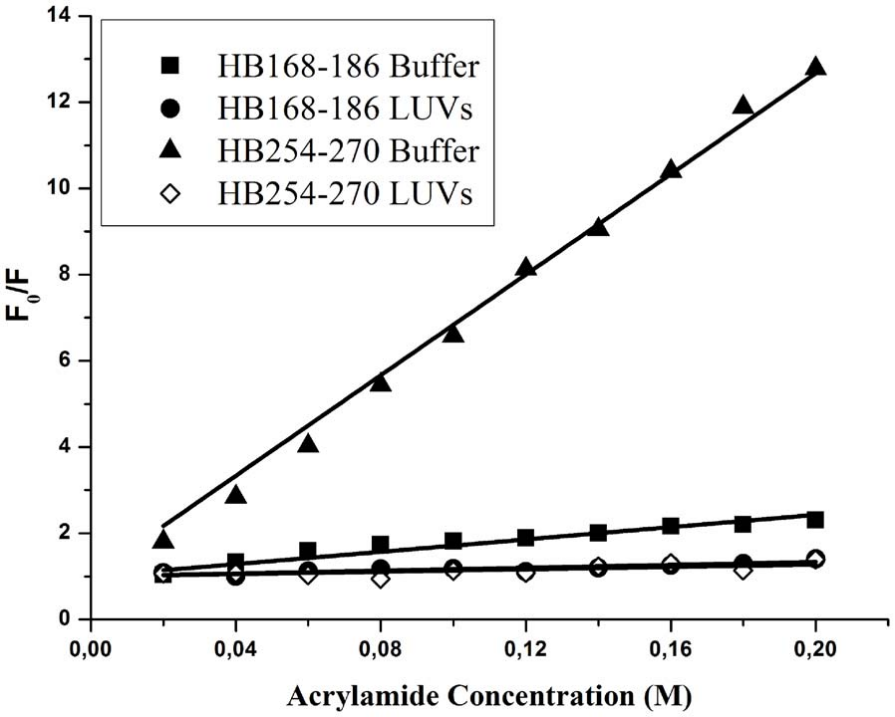
Quenching of tryptophan/tyrosines by Acrylamide. Stern-Volmer plots of acrylamide quenching of HB168–186 and HB254–270 in buffer (closed symbols) and in LUVs (open symbols).

The observed changes in the characteristics of the tryptophan/tyrosine emission upon binding of peptides HB168–186 and HB254–270 to lipid vesicles indicate their insertion into the hydrophobic region of the bilayers. We also studied the accessibility of the tryptophan/tyrosine residues of membrane-bound peptides towards acrylamide, a neutral, water-soluble, highly efficient quenching molecule, which is unable to penetrate into the hydrophobic core of the lipid bilayer. The more deeply a tryptophan/tyrosine residue is buried, the less strongly it can be quenched by acrylamide. Stern-Volmer plots for the quenching of tryptophan by acrylamide, recorded in the absence and presence of lipid vesicles, are depicted in Figure 6. Fluorescence of tryptophan/tyrosine decreased in a concentration-dependent manner by the addition of acrylamide to the peptide solution both in the absence and presence of liposomes, without other effects on the spectra. However, we can observe a substantial difference between the two peptides. In the presence of liposomes, a great decrease in fluorescence intensity was evident for HB254–270, thus revealing that tyrosine residues are less accessible to the quencher in the presence of LUVs. In fact, the values for K_sv_ were lower (Table 3) in LUVs, suggesting that tyrosines were more buried in the bilayers, becoming more inaccessible for quenching by acrylamide. From Figure 6, it is evident that we can observe for the peptide HB168–186 a low accessibility to the quencher both in absence and presence of liposomes, indicating that the peptide has a significant tendency to aggregate also in an aqueous solution.

**Table 3:** Stern-Volmer (Ksv) quenching constant calculated from the equation F_0_/F = 1+Ksv [Q] for HB168–186 and HB254–270.

	HB168–186	HB254–270
**K_sv_ (M^−1^) in buffer**	8.3±0.2	71.1±1.1
**K_sv_ (M^−1^) in LUVs**	6.4±0.4	8.5±0.1

### ESR Results

**Figure 7:**
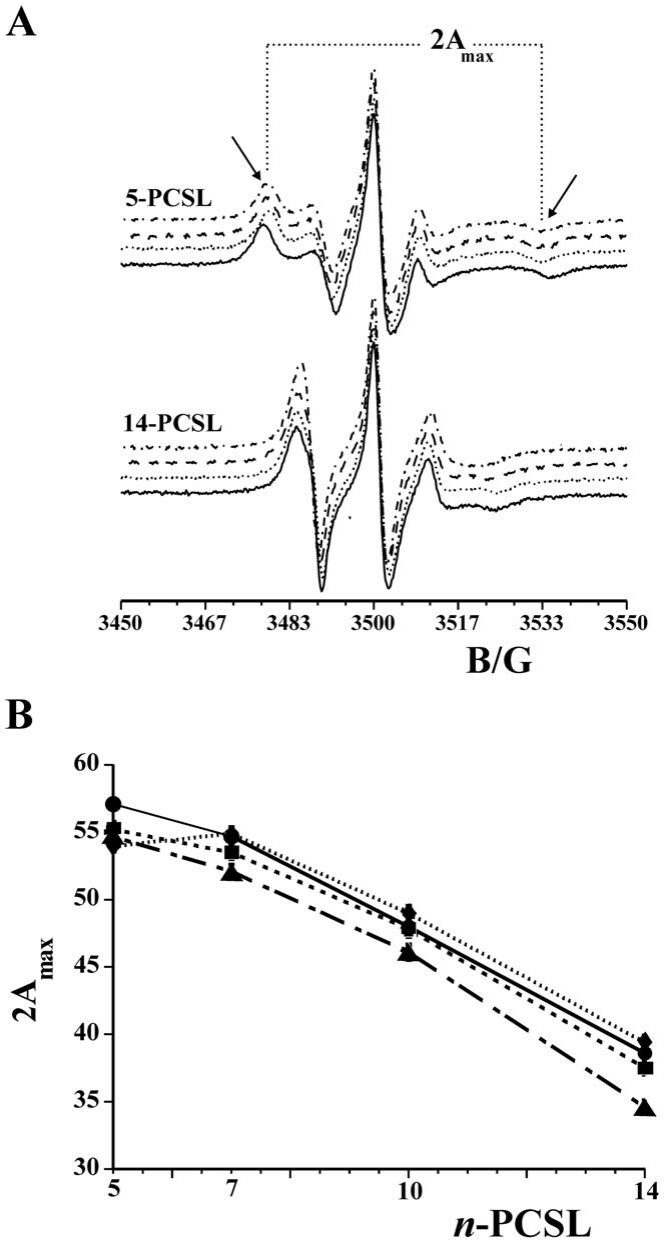
ESR **Results** **.** (**A**) ESR spectra of 5-PCSL and 14-PCSL spin-labels in DOPC/Chol membranes in the absence of peptides (solid line) and in the presence of HB168–186 (dotted line), HB254–270 (dashed line) and HB168–186–HB254–270 mixture (dashed-dotted line). (**B**) Dependence on spin-label position, *n*, of the outer hyperfine splittings, 2A_max_, of the *n*-PCSL in DOPC/Chol bilayers in the absence (○) and in the presence of HB168–186 (▪), HB254–270 (•) or HB168–186: HB254–270 (▴) at T = 25°C.

The ESR spectroscopy, by using spin-labelled substances (peptides and/or lipids) has been proved to give substantial information on the interaction of peptide deriving from viral fusion glycoproteins with lipid membranes [38]–[41]. In the present work, the association of the two peptides, HB168–186 and HB254–270, with lipid bilayers was investigated by analysing changes in ESR spectra of spin-labelled phospholipids. The samples investigated were phosphatidylcholine spin-labelled at different positions, *n*, in the s*n*-2 chain (*n*-PCSL, *n* = 5, 7, 10, 14) incorporated in DOPC/Chol membranes (3/2), in the presence of the peptides. Preliminarily, the spectra in the absence of the peptides were registered. Inspection of Figure 7A (solid lines) shows that all the spectra present a clearly defined axially anisotropic lineshape, an evidence that, due to the high cholesterol content, the DOPC/Chol bilayer is in the liquid-ordered state [42]. In an attempt to quantitatively analyse the spectra, the outer hyperfine splitting, 2A_max_, was calculated. The 2A_max_ variation, shown in Figure 7B, is an evidence of the flexibility gradient in segmental acyl chain mobility [38], [43], indicating that the lipid bilayer presents a rigid surface and relatively fluid interior [44]–[47].

Association of peptides to the lipid bilayer causes a significant variation in the ESR spectra of spin-labelled phospholipids. In Figure 7A, ESR spectra of 5-PCSL and 14-PCSL in DOPC/Chol bilayers, in the presence of HB168–186, HB254–270 and HB168–186:HB254–270 mixture at a lipid/peptide weight ratio of 1/1, are also reported. The presence of two peptides induces significant changes in the spin-label ESR spectra, which are mainly detectable from the low- and high-field component position and lineshape. In an attempt to quantify this evidence, the 2A_max_ values were determined. Figure 7B shows the dependence of these parameters on chain position, *n*, for the *n*-PCSL spin-labels in DOPC/Chol membranes, in the absence and in the presence of the peptides. In all cases, the flexibility gradient with the chain position of the lipid bilayer membranes is preserved. However, inspection of the figure reveals a significantly different behaviour of the lipid chain mobility in the co-presence of the two peptides.

In fact, addition of HB168–186 or HB254–270 significantly reduces the 2A_max_ value of 5-PCSL. In both cases, no changes in the spectra of the spin-labels bearing the nitroxide group in the more interior positions were observed. Strikingly, addition of the HB168–186:HB254–270 mixture results in a strongly 2A_max_ decrease for all the considered spin-labels. These results show a cooperation of the peptides in perturbing the bilayer microstructure in that only in the presence of both of them the increase of segmental mobility propagates along the whole acyl chains. Thus, the contemporary interaction of both peptides with the lipid membrane surface effectively perturbs the local order and dynamics of the lipid leaflet they come in contact with.

### Secondary structure in lipid bilayers

**Figure 8:**
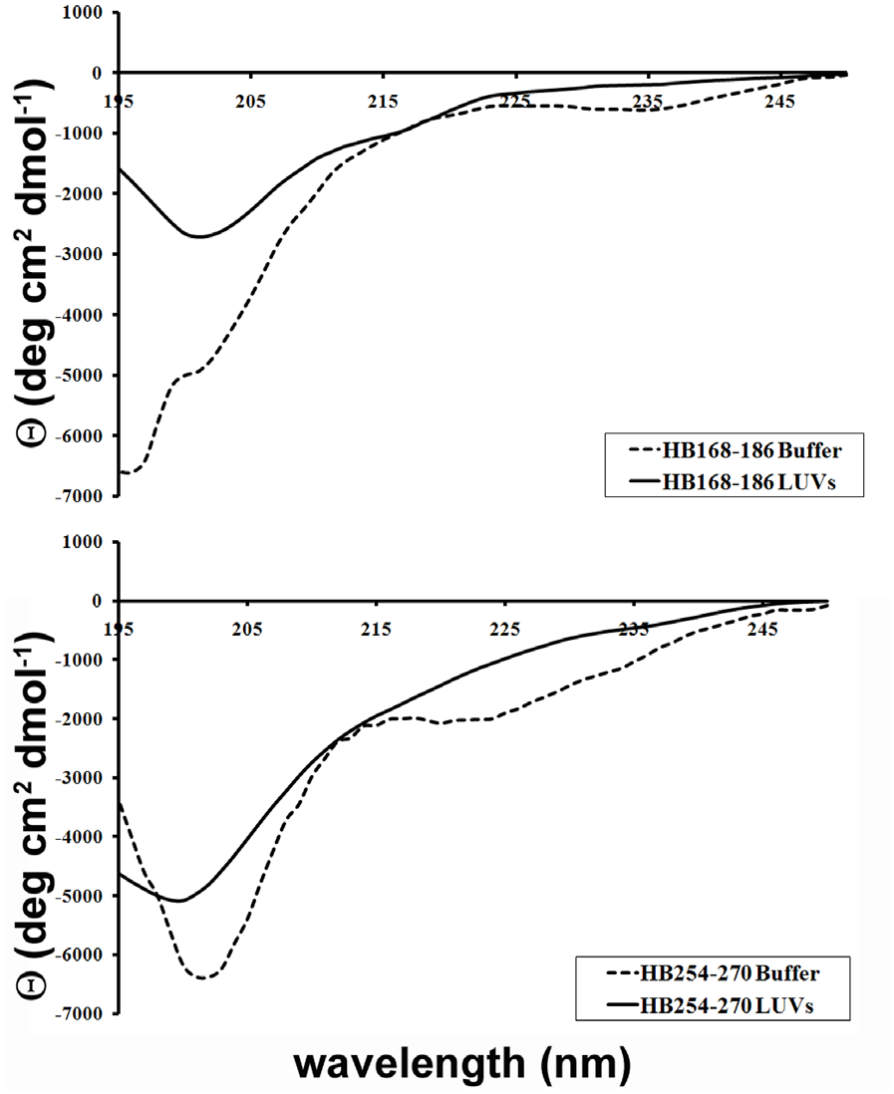
Secondary structure in lipid bilayers. CD spectra of HB168–186 and HB254–270 in buffer and in DOPC/Chol LUVs.

The CD spectra of the peptides in buffer and bound to LUVs made of DOPC/Chol are shown in Figure 8. The binding of the peptides to the membrane bilayer did not affect their structure. The spectra are indicative of random conformation for both peptides.

## Discussion

Membrane fusion is of fundamental importance in the biological life of a cell and is of particular interest during enveloped virus infections. A common feature of all fusion events is the involvement of the fusion peptide in these phenomena and the key role played by lipids in the conformational changes leading to the interaction of the fusion peptide with target membranes, and in the membrane deformation following the initial peptide-membrane interactions. Extensive research data on viral membrane fusion proteins have disclosed the existence of several protein domains involved in the viral fusion process. It is, thus, an accepted view the presence of different membrane-active regions in viral fusion glycoproteins, although their biological function is still unclear. It has been hypothesised that these regions may have a different role on either pore formation and stabilization, viral budding or both of them.

In the present work we have investigated the role played by the two fusion loops of HSV-1 gB in the mechanism of membrane fusion. In particular, three important issues were addressed: the ability of peptide analogues of the fusion loops to induce fusion of liposomes, their ability to interact with the membrane bilayer when alone and when used in equimolar concentrations and the role of the target membrane composition. For herpesviruses, cholesterol is thought to play a key role, in fact, lipid raft may act as a platform allowing cell entry and potential coreceptors clustering [32] or cholesterol may modulate the HSV entry process regardless of its ability to promote lipids microdomains [48]. In this work evidences have been obtained indicating the ability of the two fusion peptide to induce fusion of liposomes in vitro, their ability to strongly interact with a membrane bilayer containing cholesterol and finally their different mode of interaction with the bilayer indicating that although being both involved in the fusion mechanism they may play a different role.

The two fusion loops constitute a structural subdomain wherein key hydrophobic amino acids form a ridge that is supported on both sides by charged residues. The two charged residues located on both sides of the ridge represent a novel feature of viral fusion peptides and probably enhance the ability of the hydrophobic residues to interact with target membranes and to promote fusion.

The analysis of the location of the fusion loops into the three-dimensional structure of gB points out that the hydrophobic residues do not appear to be able to insert deeply into a target membrane. The hydrophilic residues on either side of the hydrophobic ridge may help to stabilize insertion of gB into cholesterol enriched membranes or more generally membranes mimicking lipid rafts, but both fusion loops seem unable to deeply penetrate into the hydrophobic core of the bilayer.

The fusion ability of the two peptides was analysed using LUVs with different compositions. Membrane components, such as anionic lipids (DOPG), unsaturated phospholipids (DOPC, POPC), sphingolipid (SM) and cholesterol (Chol) were used in this study. Lipid raft formation occurs by spontaneous aggregation of certain naturally occurring lipids that aggregate in the plane of the membrane and are characterized by a higher degree of molecular order and by being thicker than the surrounding liquid-disordered lipids in the membrane. To investigate lipid raft characteristics in model membranes, Chol and SM are commonly combined with phospholipids having unsaturated and therefore kinked fatty acyl chains, such as POPC. Our results are consistent with previous studies showing the specific association of gB with cholesterol-rich rafts [32]. We found that gB peptides associate with lower fusion ability with liposomes in the absence of cholesterol. Our data further suggest that the cholesterol dependence of gB is not necessarily dependent on a protein receptor in rafts, but because cholesterol itself enhances insertion of the fusion loops. In Semliki Forest virus fusion protein E1, a class II fusion protein that inserts preferentially into membranes enriched in cholesterol and sphingolipid [49], a point mutation in the loop adjacent to the fusion loop confers increased cholesterol independence [50], [51].

Lipid mixing, inner-monolayer and leakage are three independent experiments which describe different processes during the interaction of peptides with liposomes. In particular, the lipid mixing experiment evidences the fusion of both the inner and outer monolayer, the inner monolayer experiment evidences the eventual presence of fusion of the inner monolayer, while the leakage describes the pore formation. Results were consistent among the three experiments for both peptides, supporting the hypothesis that the two peptides induce fusion of inner and outer monolayers but not formation of pores whenever used alone or in equimolar concentrations. The present results can be used as qualitative indicators of bilayer perturbation due to its interaction with the peptides and their superficially positioning. We have not detected any significant pore formation; in fact, vesicle fusion events were not accompanied by leakage of the aqueous contents of the vesicle as also reported for other peptides in a study published by Thoren et al. [52].

Another novel feature revealed in the present work is the demonstration of oligomerization concomitant to fusion taking place. Although a correlation between penetration and fusion awaits further experimental support, our results suggest that oligomerization is another parameter related to the nature of the interaction of the peptide with the target membrane and plays a key role in governing fusion activity. The peptide HB168–186 presents a high tendency to oligomerize both in aqueous solution and inside the membrane. On the contrary, the peptide HB254–280 is not oligomerized in aqueous solution but it is able to oligomerize when interacting with the membranes.

Tryptophan and tyrosine side chains are often found at the interface between charged phospholipids and hydrophobic fatty acid chains of lipid membranes. The K_p_ values for the two peptides are reported in Table 2. Since K_p_ values are all of the same order (10^4^), we concluded that the peptides have similar membrane-binding affinities. The values of K_p_ obtained are within the range of those obtained for membrane-permeating bioactive peptides such as mellitin and its derivatives [53], the Staphylococcus δ-toxin [54], the antibiotic dermaseptin [55], pardaxin analogues [56]. All the results obtained support a shallow insertion of the two peptides that may expand the head-group region of one of the monolayers and generate elastic stresses that are released by bilayer deformation. Our results demonstrate that these two gB peptides bind and interact with membranes and could thus be directly involved in merging of the viral and cellular membranes and might work cooperatively with other membrane active regions present on herpesvirus glycoproteins to boost the fusion process. These results improve the current understanding of the critical features required for viral fusogenic peptides to cross the membrane bilayer and seem promising for the development of new carrier peptides that could exploit viral membranotropic peptides for intracellular delivery [57]–[59].

HSV-1 might have several domains involved in the fusion process which either directly or indirectly, might interact with biological membranes, contributing to the viral envelope and cell membrane merging. Although the detailed mechanism of herpesvirus entry remains unclear, some important insights into the interplay of the proteins involved in membrane fusion have been gained recently, but there are still many unsolved questions such as if the two fusion loops are the only HSV fusion peptide; what triggers them to be inserted into the target membrane, what is the role of gH in fusion and if fusion domains present in gH are directly involved in fusion.

## Materials and Methods

### Materials

The Phospholipids: 1-palmitoyl-2-oleoyl-*sn*-glycero-3-phosphocholine (POPC), dioleoyl phosphatidylcholine (DOPC), dioleoyl phosphatidylglycerol (DOPG), N-octadecanoyl-D-*erythro*-sphingosylphosphorylcholine (SM), the fluorescent probes N-(7-nitro-benz-2-oxa-1,3-diazol-4-yl)phosphatidylethanolamine (NBD-PE) and N-(Lissamine-rhodamine-B-sulfonyl) phosphatidylethanolamine (Rho-PE) were purchased from Avanti Polar Lipids (Birmingham, AL, USA), while cholesterol (Chol) and Triton- ×100 were from Sigma (St. Louis, MO, USA). All other reagents were of analytical grade. Spin-labelled phosphatidylcholines (n-PCSL) with the nitroxide group at different positions, n, in the sn-2 acyl chain were synthesized as described by Marsh [60]. The spin-labels were stored at −20°C in ethanol solutions at a concentration of 1 mg/mL.

### Peptide synthesis

Peptides were synthesised using standard solid-phase-9-fluorenylmethoxycarbonyl (Fmoc) method as previously reported [25]. All purified peptides (purity higher than 98%) were obtained with good yields (30–40%). Table 1 shows the sequences of all the synthesized peptides. Peptide stock solutions were prepared in buffer with 2% dimethyl sulfoxide (DMSO).

### Liposome preparation

Large unilamellar vesicles (LUVs) consisting of DOPG, DOPG/Chol (3/2), DOPC, DOPC/Chol (3/2), POPC/Chol (3/2), POPC/Chol/SM (4/1/1), POPC/Chol/SM (2/1/1) and POPC/Chol/SM (1/1/1), and when necessary containing Rho-PE and NBD-PE, were prepared according to the extrusion method of Hope et al. [61] in 5 mM HEPES, 100 mM NaCl, pH 7.4. Lipids were dried from a chloroform solution with a nitrogen gas stream and lyophilized overnight. For fluorescence and circular dichroism experiments, dry lipid films were suspended in buffer by vortexing for 1 h; then the lipid suspension was freeze-thawed 6 times and extruded 20 times through polycarbonate membranes with 0.1 µm diameter pores to produce LUVs. Lipid concentrations of liposome suspensions were determined by phosphate analysis [62]. For ESR spectroscopy, multi-lamellar vesicles (MLVs) were prepared. In these samples, 1% (wt/wt) of the spin-label, dissolved in ethanol, was added to the lipid mixture in organic solvent before drying. MLVs suspensions were obtained by the same procedure described above, excluding the extrusion step.

### Lipid mixing assays

Membrane lipid mixing was monitored using the resonance energy transfer assay (RET) reported by Struck et al. [63]. The assay is based on the dilution of the NBD-PE (donor) and Rho-PE (acceptor). Dilution due to membrane mixing results in an increase in NBD-PE fluorescence. The change in donor emission was monitored as aliquots of peptides were added to vesicles. Vesicles containing 0.6 mol % of each probe were mixed with unlabelled vesicles at a 1∶4 ratio (final lipid concentration 0.1 mM). Small volumes of peptides in dimethylsulfoxide (DMSO) were added; the final concentration of DMSO in the peptide solution was no higher than 2%. The NBD emission at 530 nm was followed with the excitation wavelength set at 465 nm. A cut off filter at 515 nm was used between the sample and the emission monochromator to avoid scattering interferences. The fluorescence scale was calibrated such that the zero level corresponded to the initial residual fluorescence of the labelled vesicles and the 100% value corresponding to complete mixing of all lipids in the system was set by the fluorescence intensity of vesicles upon the addition of Triton X-100 (0.05% v/v) at the same total lipid concentrations of the fusion assay. Lipid mixing experiments were repeated at least three times and results were averaged. Control experiments were performed using a scrambled peptide and DMSO. All the experiments were performed at 37°C.

### Inner-monolayer phospholipid-mixing (fusion) measurement

Peptide-induced phospholipid-mixing of the inner monolayer was measured by a modification of the phospholipid-mixing measurement reported elsewhere [64]. The concentration of each of the fluorescent probes within the liposome membrane was 0.6% mol. LUVs were treated with sodium dithionite 100 mM (from a stock solution of 1 M dithionite in 1 M TRIS, pH 10.0) to completely reduce the NBD-labelled phospholipid located at the outer monolayer of the membrane, for approximately 1 h on ice in the dark. Sodium dithionite was then removed by size exclusion chromatography through a Sephadex G-75 50 DNA Grade filtration column (GE Healthcare) eluted with a buffer containing 10 mM TRIS, 100 mM NaCl, and 1 mM EDTA, pH 7.4.

### Measurements of ANTS/DPX leakage

The ANTS/DPX assay [34] was used to measure the ability of the peptide to induce leakage of ANTS/DPX pre-encapsulated in liposomes. Details of this assay can be found elsewhere [35]. To initiate a leakage experiment, the peptide, in a stock solution at pH 7.4 containing 5 mM Hepes and 100 mM NaCl, was added to the stirred vesicle suspension (0.1 mM lipid) at 37°C.

### Thioflavin T assays for peptide aggregation

Peptide aggregation was assayed using Thioflavin T (ThT). ThT associates rapidly with aggregated peptides giving rise to a new excitation maximum at 450 nm and an enhanced emission at 482 nm [36]. LUVs in 100 mM NaCl, 10 mM Tris–HCl, 25 µM ThT, pH 7.4 (final phospholipid concentration of 0.1 mM) were titrated with a peptide concentration of 1, 4, 8, 12, 16, 20 µM. Fluorescence was measured before and after the desired amount of peptide was added into the cuvette using a Varian Cary Eclipse fluorescence spectrometer at 37°C. Samples were excited at 450 nm (slit width, 5 nm) and fluorescence emission was recorded at 482 nm (slit width, 5 nm). Aggregation was quantified according to the equation, %A = (F_f_−F_0_)/(F_max_−F_0_)×100, where F_f_ is the value of fluorescence after peptide addition, F_0_ the initial fluorescence in the absence of peptide and F_max_ is the fluorescence maximum obtained immediately after peptide addition. Kinetic data were obtained at a concentration of 4 µM.

### Tryptophan and Tyrosine fluorescence measurements

Emission spectra of the peptides (1 µM) containing the tryptophan and tyrosine residue in the absence or presence of target vesicles (DOPC/Chol 1/1) were recorded: between 300 and 400 nm with an excitation wavelength of 295 nm for the peptide HB168–186 and 290 and 350 with an excitation wavelength of 274 nm for the peptide HB254–270.The degree of peptide association with lipid vesicles was measured by adding lipid vesicles to 1 µM peptides and the fluorescence intensity was measured as a function of the lipid/peptide molar ratio, in three to four separate experiments. The fluorescence values were corrected by taking into account the dilution factor corresponding to the addition of microliter amounts of liposomes and by subtracting the corresponding blank. The lipid/peptide molar ratio was 200∶1.

The binding of hydrophobic peptides to membranes can be described as a partition equilibrium: X_b_ = K_p_C_f_ where K_p_ is the apparent partition coefficient in units of M^−1^, X_b_ is the molar ratio of bound peptide per total lipid and C_f_ is the equilibrium concentration of the free peptide in solution, as previously suggested by Schwarz et al. [65]. F_∞_ was obtained by extrapolation of a double reciprocal plot of the total peptide fluorescence vs the total lipid concentration in the outer leaflet, i.e. 1/F vs 1/0.6C_L_. The fraction of membrane-bound peptide, f_b_, was determined by the formula f_b_ = (F−F_0_)/(F_∞_−F_0_), where F represents the fluorescence of peptide after the addition of the vesicles and F_0_ represents the fluorescence of the unbound peptide. f_b_ allowed us to calculate the equilibrium concentration of free peptide in the solution, C_f_, and the extent of peptide binding X_b_. Assuming that the peptides were initially partitioned only over the outer leaflet of the SUV (60% the total lipid) [66], values of X_b_ were corrected as follows: X_b_^*^ = X_b_/0.6.

The curve resulting from plotting X_b_^*^ versus the concentration of the free peptide, C_f_, is the binding isotherm.

### Tryptophan and Tyrosine quenching by Acrylamide

Aliquots of a 4 M solution of the water-soluble quencher were added to the solution containing the peptide (1 µM) in the absence or presence of liposomes at a peptide/lipid molar ratio of 1/200. The maximal concentration of acrylamide is 0.2 mmol/ml. Tryptophan fluorescence was measured with an excitation wavelength of 295 nm, to reduce acrylamide absorbance (and the resulting inner filter effect), and emission at a wavelength of 340 nm, to eliminate interference from the Raman band of water [67] and tyrosine fluorescence was measured with an excitation wavelength of 274 nm, and emission at a wavelength of 305. The data were analyzed according to the Stern-Volmer equation [68], F_0_/F = 1+K_sv_ [Q], where F_0_ and F represent the fluorescence intensities in the absence and the presence of the quencher (Q), respectively, and K_sv_ is the Stern-Volmer quenching constant, which is a measure of the accessibility of tryptophan to acrylamide. Acrylamide does not significantly partition into the membrane bilayer [67], and the value for K_sv_ is a reliable reflection of the bimolecular rate constant for collisional quenching of the aromatic residues present in the aqueous phase. Accordingly, K_sv_ is determined by the amount of non-vesicle-associated free peptide as well as the fraction of the peptide residing at the surface of the bilayer.

### Electron Spin Resonance Spectroscopy

ESR spectra were recorded with a 9 GHz Bruker Elexys E-500 spectrometer (Bruker, Rheinstetten, Germany). The lipid suspensions to be investigated were transferred into 25 µL glass capillaries and flame sealed. The capillaries were placed in a standard 4 mm quartz sample tube containing light silicone oil for thermal stability. All the measurements were performed at 25°C. Spectra were recorded using the following instrumental settings: sweep width, 100 G; resolution, 1024 points; time constant, 20.48 ms; modulation frequency, 100 kHz; modulation amplitude, 1.0 G; incident power, 6.37 mW. Samples containing the peptides were prepared by hydrating the lipid film directly with the peptide solution in buffer. In all samples, the peptide to lipid ratio was set to 1∶1 wt/wt. In samples containing both HB168–186 and HB254–270, the total peptide to lipid ratio was maintained to 1∶1 wt/wt, while the HB168–186∶HB254–270 ratio was 1∶1 wt/wt.

Several scans, typically 16, were accumulated to improve the signal-to-noise ratio. Values of the outer hyperfine splitting, 2*A_max_*, were determined by measuring the difference between the low-field maximum and the high-field minimum, through a home-made, MATLAB-based, software routine. This parameter is a useful empirical measure of the lipid chain dynamics and order in both gel and fluid phases of lipid bilayers [44]–[46]. The main source of error on the 2*A_max_* value is the uncertainty in composition of samples prepared by mixing few microliters of mother solutions. For this reason, reproducibility of 2*A_max_* determinations was estimated by evaluating its value for selected independently prepared samples with the same nominal composition. It was found to be ±0.2–0.3 G.

### Circular dichroism spectroscopy

CD spectra were recorded using a Jasco J-715 spectropolarimeter in a 1.0 cm quartz cell at room temperature. The spectra are an average of 3 consecutive scans from 260 to 195 nm, recorded with a band width of 3 nm, a time constant of 16 s, and a scan rate of 10 nm/min. Spectra were recorded and corrected for the blank sample. Mean residue ellipticities (MRE) were calculated using the expression MRE = *Obsd*/(*lcn*), where *Obsd* is the ellipticity measured in millidegrees, *l* is the path length of the cell in cm, *c* is the peptide concentration in mol/l, and *n* is the number of amino acid residues in the peptide. Solutions of 4 µM of HB168–186 and 10 µM of HB254–270 with LUVs were prepared as described previously [31]. The measurements were performed at peptide/lipid ratios of 0.5 mol/mol.

## Supporting Information

Figure S1 **Fluorescence spectra of tryptophan and tyrosine.** Fluorescence spectra of HB168–186 and HB254–270 in buffer and in LUVs.
(TIF)
