# Supplementary figures and images for: Biophysical Characterization and Membrane Interaction of the Two Fusion Loops of Glycoprotein B from Herpes Simplex Type I Virus
Source: PLoS One. 2012 Feb 23;7(2):e32186. doi: 10.1371/journal.pone.0032186 (PMC3285657; doi:10.1371/journal.pone.0032186)

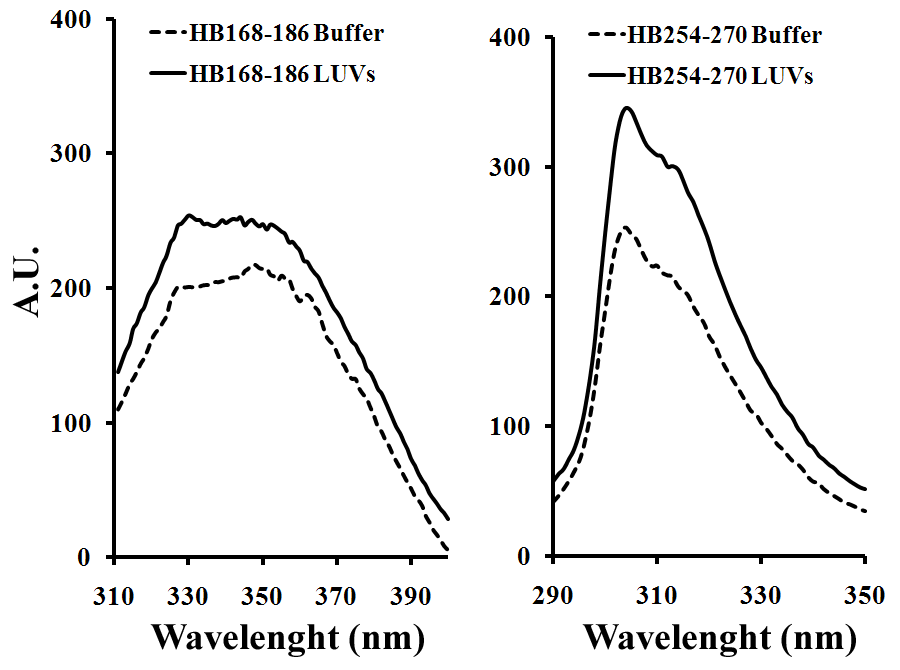

Supplement: Figure S1 — Fluorescence spectra of tryptophan and tyrosine. Fluorescence spectra of HB168–186 and HB254–270 in buffer and in LUVs. (TIF) [file pone.0032186.s001.tif]
